# The effect of ribociclib on the expression levels of *miR-141* and *CDK4/6-USP51* signaling pathway genes in *MCF-7* and *MDA-MB-231* cells

**DOI:** 10.1371/journal.pone.0309289

**Published:** 2024-08-28

**Authors:** Shayeste Sadat Baghermanesh, Mahmood Barati, Arshad Hosseini

**Affiliations:** Department of Medical Biotechnology, Faculty of Allied Medical Sciences, Iran University of Medical Sciences, Tehran, Iran; Northwest University, UNITED STATES OF AMERICA

## Abstract

**Introduction:**

Patients with breast cancer, especially triple-negative breast cancer, have a poor prognosis. There is still no effective treatment for this disease. Due to resistance to traditional treatments such as chemotherapy and radiation therapy, there is a need to discover novel treatment strategies to treat this disease. Ribociclib is a selective *CDK4/6* inhibitor. Approximately 20% of patients with HR+ breast cancer developed primary resistance to *CDK4/6* inhibitors, and more than 30% experienced secondary resistance. Since most patients experience resistance during *CDK4/6* inhibitor treatment, managing this disease is becoming more challenging. Many malignant tumors abnormally express microRNA (miR)-141, which participates in several cellular processes, including drug resistance, proliferation, epithelial–mesenchymal transition, migration, and invasion.

**Materials and methods:**

In the present study, we cultured *MDA-MB-231* and *MCF-7* cells in DMEM-F12 medium. By performing MTT assay we determined the cytotoxic effects of ribociclib on breast cancer cells, as well as determining the IC50 of it. Then, we treated the cells with ribociclib at two time points: 24 h and 72 h. After that, RNA was isolated and reverse transcribed to cDNA. Finally, we performed qRT‒PCR to evaluate how ribociclib affects the expression level of desired genes.

**Results and conclusion:**

We found that ribociclib can inhibit cell growth in a dose- and time-dependent manner. We examined the mRNA expression of 4 genes. After ribociclib treatment, the mRNA expression of *CDK6* and *MYH10* decreased (p < 0.01, p < 0.05). The mRNA expression of *CDON* increased (p<0.05), but no significant changes were observed in *ZEB1* mRNA expression. Furthermore, the qRT‒PCR results for *miR-141* showed that the expression of *miR-141* increased (p<0.01) after 72 h of treatment with ribociclib.

## Introduction

Breast cancer is the most commonly diagnosed cancer worldwide [1]. According to the levels of mRNA gene expression, breast cancer can be classified into molecular subtypes that provide insight into new treatment strategies and patient stratifications [2]. Unlike *MDA-MB-231* cells, which are more aggressive and hormone independent, *MCF-7* cells are noninvasive and have functional estrogen and *EGF* receptors [3].

In recent years, there have been increasing reports suggesting that miRNA molecular networks may provide new therapeutic targets or biomarkers. For example, using bioinformatics tools, Maryam Eini et al. suggested that *miR-802* is a prognostic biomarker for breast cancer [4]. The *miR-200* family is one of the most prominent groups of miRNAs whose expression is altered in cancer. There are three mechanisms associated with the regulation of the expression of the *miR-200* family: 1) suppressing epithelial–mesenchymal transition (EMT) and tumor metastasis via the *miR-200/ZEB1-2* axis [5], 2) reversing chemoresistance [6], and 3) inhibiting cancer stem cell self-renewal and differentiation [7]. Some studies suggest that these compounds have oncosuppressive functions, while others suggest that they have oncogenic roles [8]. In many human malignant tumors, *miR-141*, a crucial member of the *miR-200* family, is aberrantly expressed and involved in a variety of cellular processes, such as proliferation, migration, invasion, EMT, and resistance to drugs. *miR-141* can function both as a tumor suppressor and as a tumor promoter in tumorigenicity [9].

Chemoresistance and radioresistance are the primary causes of failure in treating triple-negative breast cancer (TNBC). Hong Luo et al. and previous researchers have linked this resistance to zinc-finger E-box binding home box 1 (*ZEB1*) [10]. It has been shown that *ZEB1*, which is a transcription factor involved in EMT, has an imperative role in facilitating cell migration and invasion during tumor metastasis [11, 12]. However, how *ZEB1* is stabilized in cells is unclear since it is subject to ubiquitination and degradation. As a result of screening a human deubiquitinase library, Zhicheng Zhou et al. identified *USP51* as a deubiquitinase capable of binding *ZEB1*, deubiquitinating it, and stabilizing it. *USP51* depletion inhibited the invasion of mesenchymal-like breast cancer cells by downregulating *ZEB1* protein and mesenchymal marker expression and promoting E-cadherin upregulation. On the other hand, overexpression of *USP51* led to the upregulation of *ZEB1* and mesenchymal markers in epithelial cells [13].

Cyclin-dependent kinases are serine/threonine protein kinases that bind proline and are known as CDKs [14]. Cell cycle arrest in cancer cells can be achieved by pharmacologically inhibiting *CDK4* and *CDK6*. Recent studies suggest that *CDK4/6* inhibitors influence apoptotic responses, differentiation, and cancer cell immunogenicity. Using cell-based and mouse models of breast cancer along with clinical specimens, Watt et al. demonstrated that *CDK4/6* inhibitors regulate cancer cell chromatin through widespread enhancer activation [15]. Therefore, targeting *CDK4/6* is a paradigm shift in developing anticancer therapies [16].

Cancer therapy is becoming increasingly promising with the development of effective *CDK4/6* inhibitors, such as ribociclib, palbociclib, and abemaciclib, which are already approved [16]. The third-generation *CDK4/6* inhibitor ribociclib (LEE011) inhibits the expression of *CDK4/6* with high selectivity [17]. Despite the crucial clinical benefits of *CDK4/6* inhibitors in HR+ breast cancer [18], studies have shown that approximately 20% of patients with HR+ breast cancer develop primary resistance to *CDK4/6* inhibitors [19], and more than 30% experience secondary resistance [20]. The development of resistance in almost all patients during treatment poses new challenges for managing this disease [21].

In this study, we performed experiments on *MDA-MB-231* and *MCF-7* cell lines to evaluate the effects of ribociclib on cell growth and to analyze the changes in the expression of crucial genes involved in the *CDK4/6-USP51* signaling pathway.

## Materials and methods

### Cell lines and reagents

The human triple-negative breast cancer (TNBC) cell line *MDA-MB-231* and the nontriple-negative breast cancer (NTNBC) cell line *MCF-7* were purchased from the Iranian Biological Resource Center (IBRC). It was verified that these cell lines were not contaminated with mycoplasma. The cells were cultured in Dulbecco’s modified Eagle’s minimal essential medium/nutrient mixture F-12 (DMEM/F12, BioIdea, Iran) supplemented with 10% fetal bovine serum (FBS, BioIdea, Iran) and 1% penicillin/streptomycin antibiotic (BioIdea, Iran) and incubated in a humidified 37°C incubator with 5% CO_2_.

Ribociclib (LEE011) was purchased from MedChemExpress (MCE) USA. A 1.0 mM stock solution was produced by dissolving 5 mg of LEE011 powder (M.W.: 434.54 g/mol, HY-15777, MCE) in 11.5064 ml of dimethyl sulfoxide (DMSO, DNA Biotech, Iran). The stock solution was sterilized by a 0.22 μm filter and stored at -80°C. Ribociclib was dissolved in DMSO to create a 1mM stock solution. This stock was then diluted in cell culture medium to achieve the desired test concentration of 0.1 μM. The final concentration of DMSO in the cell culture medium did not exceed 0.1% v/v for any of the ribociclib treatment conditions.

### MTT cell viability assay

The MTT assay was used to determine the cytotoxic effects of ribociclib on breast cancer cells, as well as determining the half-maximal inhibitory concentration (IC50) of ribociclib. To evaluate cell viability, *MDA-MB-231* and *MCF-7* cells in the logarithmic phase of growth were plated into 96-well plates at a density of 10,000 cells per well. After 24 h, the cells were treated with different concentrations of ribociclib ranging from 9.5–15.5 μM and 10–30 μM for *MDA-MB-231* and *MCF-7* cells, respectively, for 24, 48, and 72 h. After adding 20 μl of MTT (3-[4,5-dimethylthiazol-2-yl]-2,5 diphenyl tetrazolium bromide) reagent (5 mg/ml; Atocel, Budapest) to each well, the 96-well plates were cultured for another 4 h at 37°C. To dissolve the precipitated violet formazan, 150 μl of DMSO was added to each well after the supernatant was removed. Finally, a microplate reader (DANA-3200, Iran) was used to measure the absorbance at a wavelength of 570 nm.

### RNA isolation

To evaluate how ribociclib affects the expression level of desired genes, we seeded *MDA-MB-231* and *MCF-7* cells in 6-well plates (SPL, Korea) at a density of 300,000 cells per well. The 6-well plates were divided into two groups: the control group (without ribociclib treatment) and the treatment group. After 1 day of incubation, the cells in each well of the treatment group were treated with the half-maximal inhibitory concentration (IC50) of ribociclib according to the results of MTT assay. It was 11 μM for *MDA-MB-231* cells and 20 μM for *MCF-7* cells. The cells were treated at two time points: 24 h and 72 h. All experiments were performed in triplicate.

Total RNA was isolated using Trizol reagent (YTzol pure RNA, YektaTazhiz Azma, Iran) according to the manufacturer’s instructions. DEPC-treated water was used to dissolve RNA, and the concentration and purity of each sample were spectrophotometrically measured by using A260/A280 measurements (NanoDrop, Thermo Scientific, USA). Additionally, agarose gel electrophoresis was used to determine RNA integrity.

### cDNA synthesis and qRT‒PCR analysis

For the detection of *ZEB1*, *CDK6*, *MYH10*, and *CDON* mRNA expression, 1 μg of total RNA was reverse transcribed into first-strand cDNA using Moloney murine leukemia virus (MMLV) reverse transcriptase with oligo(dT) and random hexamers (NoteScript, Notarkib Fast cDNA Synthesis Kit, Iran) according to the manufacturer’s instructions. Then, quantitative reverse transcription polymerase chain reaction (qRT–PCR) was performed using SYBR Green master mix (RealTiQ, Notarkib, Iran), with *GAPDH* serving as an endogenous control. A QIAGEN real-time PCR cycler (Rotor-Gene Q; QIAGEN, Germany) was used, and the following three-step temperature program was applied: 95°C for 15 minutes, followed by 40 cycles of 95°C for 15 seconds for the denaturation step, 60°C for 30 seconds for the annealing step, and 68°C for 10 seconds for the extension step. Additionally, for the detection of *miR-141* expression, 1 μg of total RNA was reverse transcribed using stem‒loop primers. A miRNA cDNA synthesis kit (Notarkib, Iran) was used to synthesize cDNA. Subsequently, qRT‒PCR was performed using SYBR Green master mix (RealTiQ, Notarkib, Iran), with *U6* serving as an internal control in a Rotor-Gene Q real-time PCR cycler. The following two-step temperature program was used: 95°C for 15 minutes, followed by 40 cycles of 95°C for 15 seconds for the denaturation step and 60°C for 60 seconds for the combined annealing/extension step. For all the samples, the qRT-PCR was examined in duplicate.

Reference genes (*GAPDH* and *U6*) were used to normalize the results. The relative expression levels of *ZEB1*, *CDK6*, *MYH10*, *CDON*, and *miR-141* were evaluated based on the 2^-ΔΔCT^ method. To design the primers, we used primer design software such as Blast Primer, Oligo7 and Primer3. The sequences of primers used for qRT‒PCR are listed in Table 1.

**Table 1:** Sequences of Primers used for qRT‒PCR.

Name	Forward Primer (5’-3’)	T_m_ (°C)	Reverse Primer (5’-3’)	Tm (°C)
GAPDH	GAAGGTGAAGGTCGGAGTC	60	GAAGATGGGATGGGATTTC	58
ZEB1	CCAGCCAAATGGAAATCAGGATG	60	TTGGGCGGTGTAGAATCAGAG	59
CDK6	AAGACTGGCCTAGAGATGTTGC	62	TCCAGGTTTTCTTTGCACCT	56
MYH10	CAAACGTCAGGGAGCATCTT	58	GCGCTGGTATTCCTCTTGTT	58
CDON	TAAAGGACGGGCAGGACATT	58	CGTCGCAGGTAAAGTGTACTG	61
U6	GGATGACGCAAATTCGTGAAGC	60	CGTGGTTAGGGTCCGAGGTA	60
MiR-141	GCGCGTAACACTGTCTGGTA	60	CGTGGTTAGGGTCCGAGGTA	60

### Bioinformatics

In this study, the targets of *miR-141* were predicted using online bioinformatics tools. The targets with the highest match score to *miR-141* according to the TargetScan, miRDB, and miRWalk databases were chosen.

GeneMANIA is a fast gene network construction and function prediction tool. The results were subsequently used to predict the interactions between *USP51* and gene targets of *miR-141*.

### Statistical analysis

In this study, GraphPad Prism version 9.0.0 (GraphPad Software, San Diego, CA, USA) was used for all the statistical analyses. Student’s t tests or analysis of variance (ANOVAs) (two-way ANOVA or mixed model) followed by Tukey’s post-test were used to determine the statistical significance of the differences between the data. The responses of cells treated with ribociclib and those in the control group were compared. A difference was considered significant at *, **, ***, and **** when the P value was less than 0.05, 0.01, 0.001, and 0.0001, respectively.

### Ethics statement

Not applicable.

## Results

### Predicted target genes of *miR-141*

**Fig 1:**
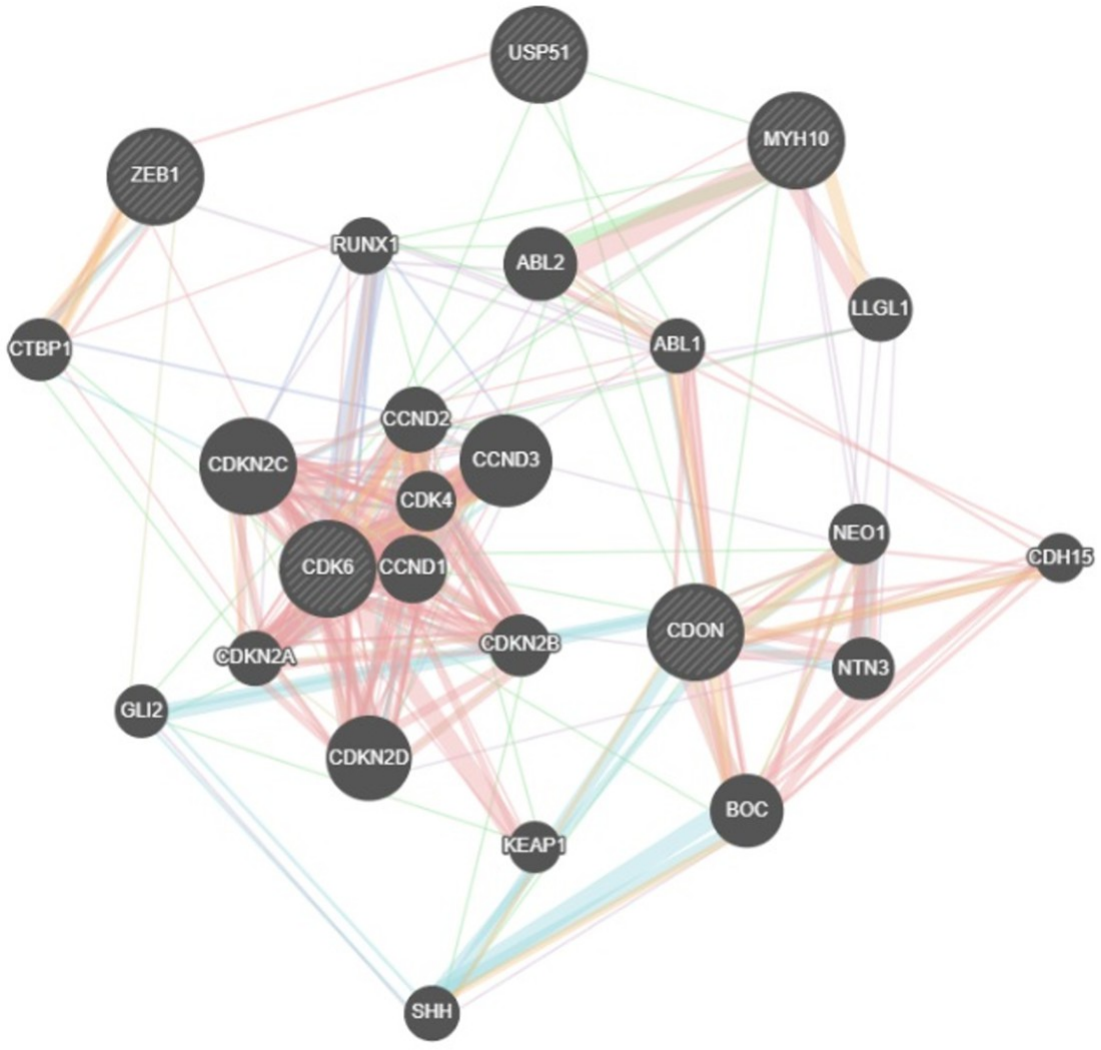
Target prediction and gene interaction. Target genes of *miR-141* that are involved in *CDK4/6-USP51* signaling pathway.

Based on searches of online databases such as TargetScan, miRWalk, miRDB, and GeneMANIA, we selected *ZEB1*, *CDK6*, *MYH10*, and *CDON* as genes involved in the *CDK4/6-USP51* signaling pathway and targets of *miR-141* (Fig 1).

### Ribociclib inhibits the growth of breast cancer cell lines in a time- and dose-dependent manner

**Fig 2:**
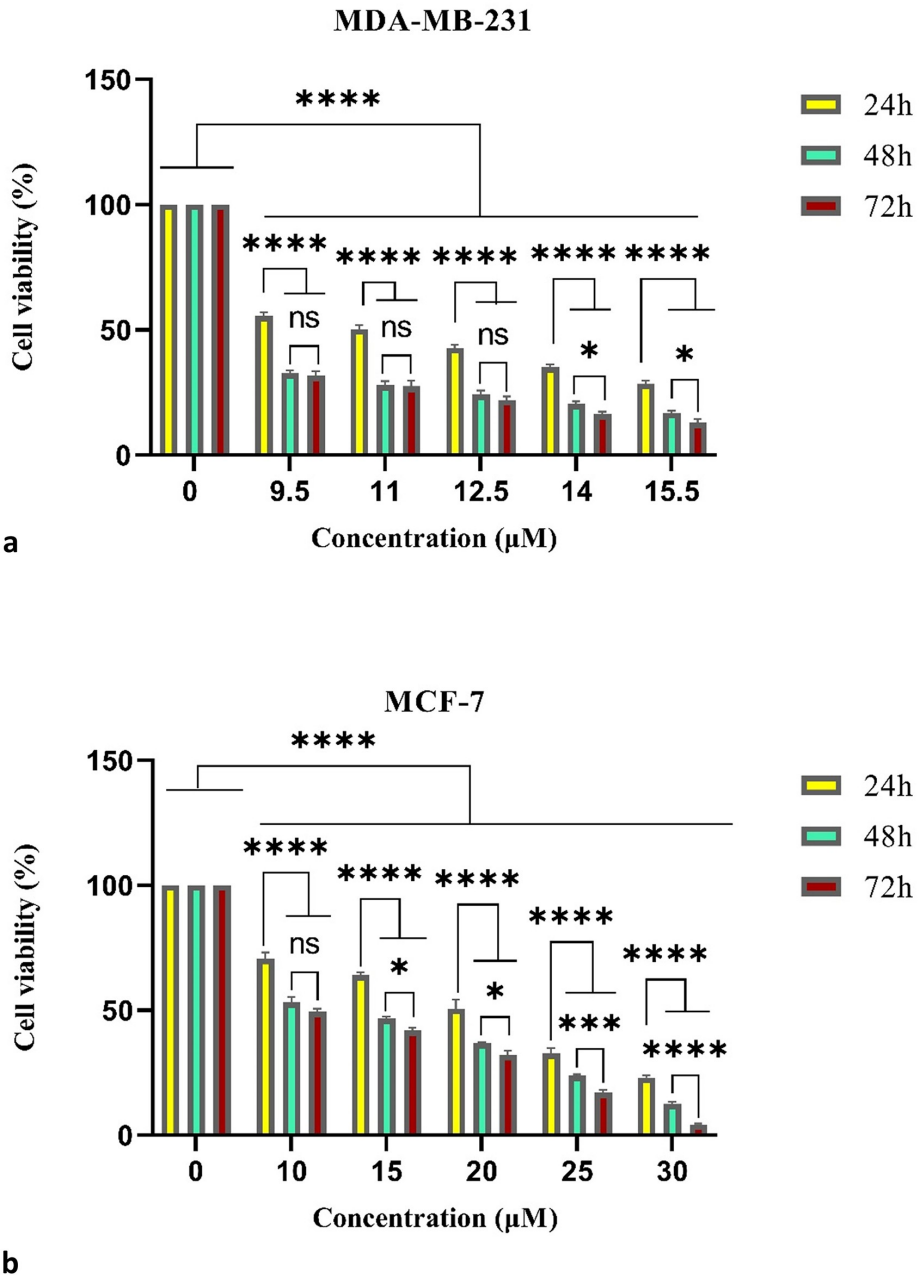
MTT assay. Cell viability was determined by MTT assay after exposure to increasing doses of Ribociclib for 24, 48, and 72 h in *MDA-MB-231* (a) and *MCF-7* (b) cell lines. ns: not significant, * p< 0.05, *** p< 0.001, **** p< 0.0001.

To investigate whether ribociclib inhibits the growth of *MDA-MB-231* (TNBC) and *MCF-7* (NTNBC) cells in a time- and dose-dependent manner, we performed an MTT assay. For this purpose, we treated *MDA-MB-231* and *MCF-7* cells with different doses of ribociclib ranging from 9.5–15.5 μM and 10–30 μM for 24, 48, and 72 h. As shown in Fig 2, cell growth decreased in both cell lines with increasing drug dose and treatment duration.

Moreover, we used the results of the MTT assay to calculate the cell viability and the IC50 value of ribociclib. These values were 11 μM and 20 μM for the *MDA-MB-231* and *MCF-7* cells, respectively.

### Ribociclib can affect the expression of genes involved in the *CDK4/6-USP51* signaling pathway

**Fig 3:**
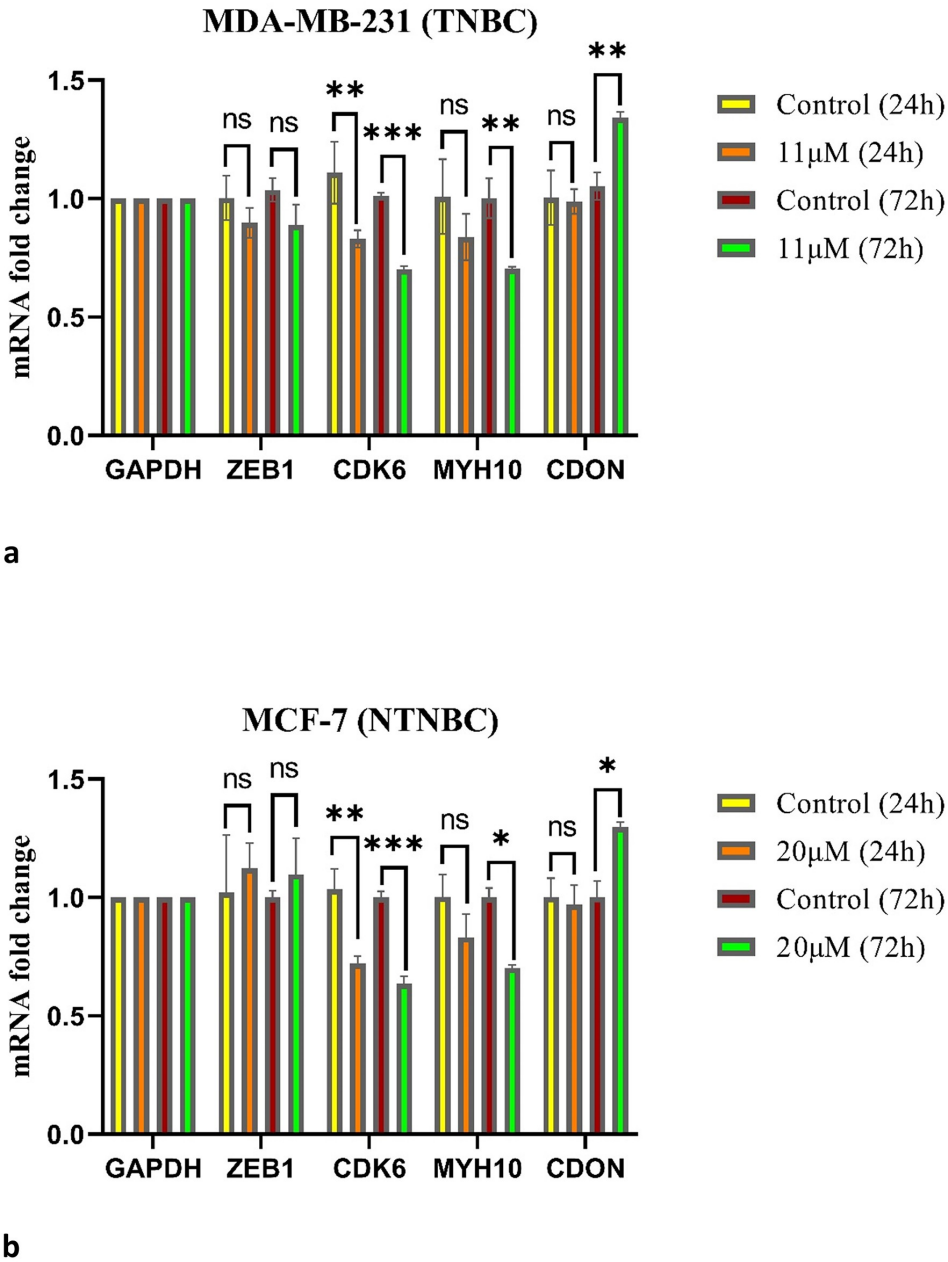
Results of qRT-PCR for target genes of *miR-141*. *ZEB1*, *CDK6*, *MYH10*, and *CDON* expression in *MDA-MB-231* cells treated with 11μM Ribociclib (a), and in *MCF-7* cells treated with 20μM Ribociclib (b) for 24 and 72 h, compared with the control group. ns: not significant, * p< 0.05, ** p< 0.01, *** p< 0.001.

**Fig 4:**
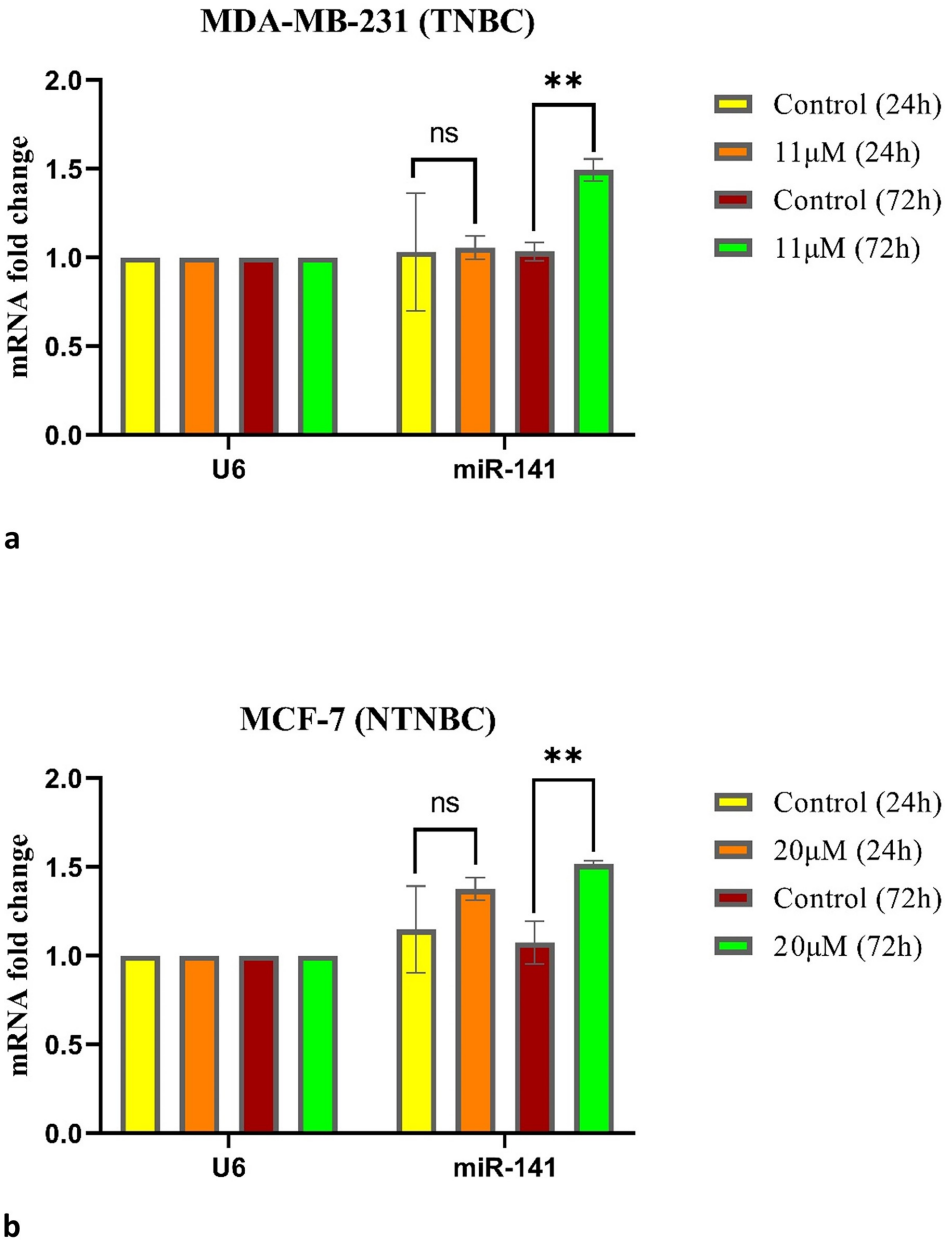
Results of qRT-PCR for *miR-141*. *miR-141* expression in *MDA-MB-231* cells treated with 11μM Ribociclib (a), and in *MCF-7* cells treated with 20μM Ribociclib (b) for 24 and 72 h, compared with the control group. ns: not significant, * p< 0.05, ** p< 0.01, *** p< 0.001.

qRT‒PCR was performed to determine whether ribociclib affects the mRNA expression of *ZEB1*, *CDK6*, *MYH10*, and *CDON*. In both cell lines, *CDK6* expression was significantly lower in the treatment group than in the control group, as shown in Fig 3. For details, see Supporting information (S1-fig 3 and fig 4 in S1 File). In addition, the expression of *MYH10* and *CDON* significantly decreased and increased, respectively, after 72 h of treatment with ribociclib. According to our results, there was no significant change in *ZEB1* mRNA expression in any of the cell lines after 72 h of treatment with ribociclib.

### Ribociclib can affect the expression of *miR-141* in breast cancer cell lines

We also evaluated the effect of ribociclib on *miR-141* expression in the TNBC and NTNBC cell lines *MDA-MB-231* and *MCF-7*. The qRT‒PCR results demonstrated that compared with no treatment, ribociclib had no effect on these cells after 24 h but significantly increased the expression of *miR-141* in cells treated with ribociclib for 72 h (Fig 4). For details, see Supporting information (S1-fig 3 and fig 4 in S1 File).

## Discussion

As TNBC is a refractory type of cancer, there is great resistance to existing treatments. Traditional therapies such as chemotherapy, radiotherapy, or combined endocrine therapy are no longer adequate for controlling cancer. Therefore, there are still many original targets to be explored in the future. The FDA has approved *CDK4/6* inhibitors, and their effectiveness in various cancers has been validated both in preclinical and clinical studies. Ongoing clinical trials have demonstrated the effectiveness of ribociclib in only a fraction of TNBC patients, while the majority of these trials have not been successful [22, 23]. With the use of drugs such as palbociclib, ribociclib, and abemaciclib, the development and design of effective *CDK4/6* inhibitors are increasingly being proven to be promising cancer treatments. Ribociclib and palbociclib have similar structures and functions; however, compared with palbociclib, ribociclib binds to *CDK4* and *CDK6* more selectively and is less toxic to the bone marrow [24–26]. Notably, the undeniable success of ribociclib in treating breast cancer also suggests that this drug could have clinical benefits in the treatment of other cancers [27, 28]. A number of clinical trials involving this drug are underway, targeting tumor indications such as cancer of the ovary and endometrium, liposarcoma, glioblastoma, central nervous system carcinoma, melanoma, and prostate cancer [28, 29]. Therefore, we chose ribociclib for this study based on the reasons outlined above.

The MTT assay results indicated that ribociclib inhibits the growth of *MDA-MB-231* and *MCF-7* cells. Specifically, at 15.5 μM for *MDA-MB-231* cells and 30 μM for *MCF-7* cells, the cells nearly died out. We treated these cells with increasing doses of ribociclib for 24, 48, or 72 h. We observed that as the drug dose and treatment duration increased, the percentage of cell viability decreased. Therefore, our results support the theory that ribociclib has an antiproliferative impact on breast cancer cells [30]. There is increasing evidence that *ZEB1* plays a role in the initiation and progression of breast cancer [12, 31]. It is therefore hoped that by identifying the signaling pathways that regulate *ZEB1* stabilization, anticancer therapies can be improved. A study by Zhen Zhang et al. revealed that inhibiting *CDK4/6* activity reduced *ZEB1* protein stability and inhibited breast cancer EMT. They found that *CDK4/6* phosphorylate and activate the deubiquitinase *USP51*, which is necessary for *ZEB1* deubiquitination and stabilization. In conclusion, the *CDK4/6-USP51-ZEB1* axis is a key regulatory pathway in breast cancer metastasis that can be exploited for diagnostic and therapeutic purposes in the future [13].

**Fig 5:**
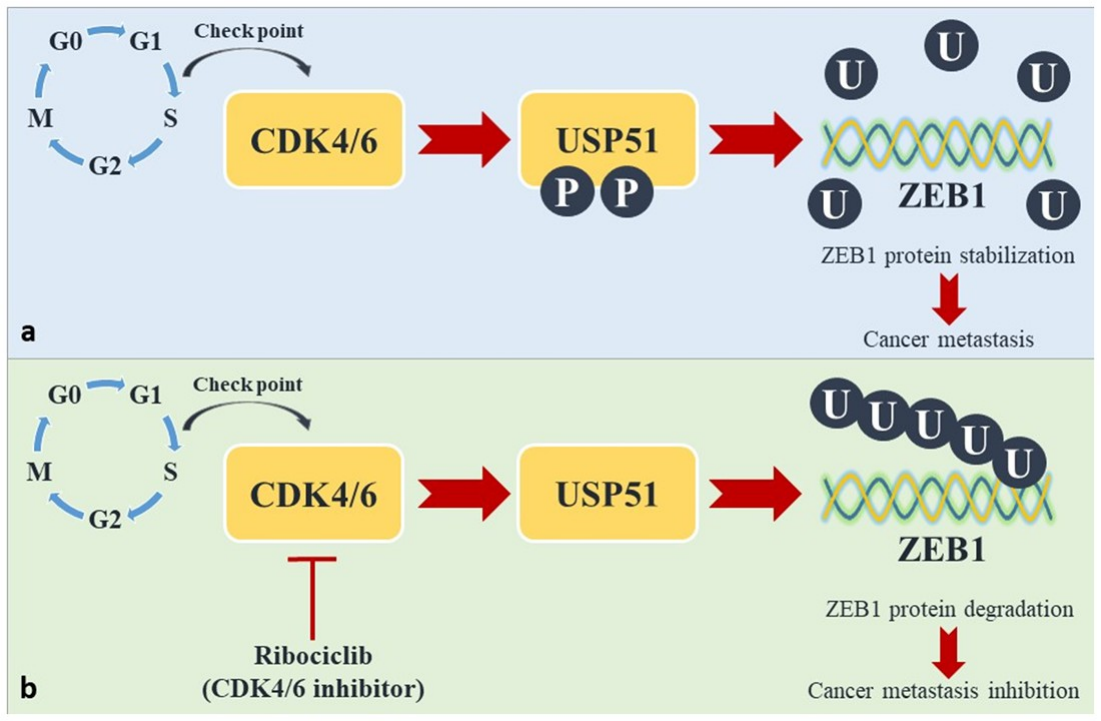
Schematic model of the *CDK4/6-USP51-ZEB1* axis. *ZEB1* protein stabilization (a). *ZEB1* protein degradation (b).

Fig 5 shows a schematic model of the *CDK4/6-USP51-ZEB1* axis. As mentioned above, due to the importance of the *CDK4/6-USP51* signaling pathway in the treatment of advanced breast cancer, we selected this signaling pathway for this study to investigate whether ribociclib affects the mRNA expression of genes involved in this pathway. As expected, qRT‒PCR analysis revealed that *CDK6* mRNA expression was significantly decreased in both cell lines after 24 h, but no significant changes were observed in the mRNA expression of *ZEB1*. Since *ZEB1* is a protein that plays a crucial role in the *CDK4/6-USP51* signaling pathway, we anticipated that its expression would decrease under treatment with ribociclib. As shown in Fig 5, *ZEB1* is located downstream of *CDK6*; thus, we hypothesized that ribociclib may affect *ZEB1* expression at the translational level. Due to the lack of western blotting analysis data, our results cannot confirm the effect of ribociclib on *ZEB1* expression in breast cancer cell lines. According to a study conducted in 2020, Zhen Zhang et al. verified that ribociclib promotes the degradation of the *ZEB1* protein in a ubiquitin‒proteasome-dependent manner in *SUM-159* and *MDA-MB-231* cells [13]. Their results strengthen our hypothesis.

In line with our results, the results of a recent study by Edris Choupani et al. showed that *CDK6* expression in *MDA-MB-468*, *MDA-MB-231*, and *MCF-7* cells treated with enzalutamide or ribociclib was significantly lower than that in the control group [32].

Based on their target mRNAs, miRNAs can function either as oncogenes or tumor suppressors. Several studies have linked the abnormal expression of miRNAs to cancer invasion, metastasis, and chemotherapy resistance. In this way, miRNA expression profiles are considered biomarkers for breast cancer prognosis and prediction [33, 34]. It has been shown that *hsa-miR-141* overexpression inhibits breast, colorectal, and pancreatic cancer invasion and migration [35–37]. There is also evidence that *miR-141* regulates colorectal, lung, gastric, liver, prostate, and renal cancer cell growth and metastasis [35, 38–42]. In a study published by Ping Li et al., *miR-141* was found to be downregulated in breast cancer tumor tissues compared to matched surrounding tissues. They also found that downregulation of *miR-141* expression was associated with *PCNA*, *Ki67*, and *HER2* expression; tumor involvement; and tumor stage. Additionally, *miR-141* overexpression inhibited the proliferation, migration, and invasion of breast cancer cells in vitro [43].

In the present study, to investigate the effect of ribociclib on *miR-141* expression, we treated *MDA-MB-231* and *MCF-7* cells with ribociclib and measured the expression of *miR-141* before and after treatment with ribociclib by qRT‒PCR. Our results showed that ribociclib significantly increased the expression of *miR-141* after 72 h of treatment.

In summary, we demonstrated that ribociclib potentially affects the mRNA expression of several effective genes in the *CDK4/6-USP51* signaling pathway, as well as the expression of *miR-141* in the *MDA-MB-231* and *MCF-7* cell lines. However, further studies are needed to determine how ribociclib affects other genes and other breast cancer cell lines, as well as normal breast cell lines such as *MCF10-A*.

## Conclusions

In conclusion, our study does not present a groundbreaking discovery, but it offers valuable contributions to the field of breast cancer research. Notably, we provided the first insights into ribociclib’s influence on specific genes (ZEB1, CDON, and MYH10) within the CDK4/6-USP51 signaling pathway, an area previously unexplored. It is important to emphasize that our findings suggest potential effects of ribociclib on certain gene expressions, which may be relevant to its mechanism of action. However, further studies are required to establish its efficacy as an antineoplastic drug.

## Future directions and need for additional studies

While our study provides initial insights into ribociclib’s effects on gene expression in breast cancer cells, further research is essential. Key areas for investigation include:

1. In vivo experiments to assess efficacy and toxicity
2. Combination studies with other therapeutic agents
3. Investigations into drug resistance mechanisms
4. Analyses of effects on different cancer cell lines and subtypes
5. Exploration of additional molecular pathways affected by ribociclib
6. Clinical correlations using patient tumor samples
7. Long-term effects on cell phenotype and gene expression

These studies would enhance our understanding of ribociclib’s mechanisms of action, its potential efficacy across different breast cancer subtypes, and its optimal use in clinical settings, addressing the critical need for effective therapies for resistant breast cancers.

## Supporting information

S1 File Raw data and statistical analysis for Figs 3 and 4. (XLSX)
